# Supplementary material for: Pyruvate Dehydrogenase Kinase 1 inhibition mediated oxidative phosphorylation enhancement in cartilage promotes osteoarthritis progression
Source: BMC Musculoskelet Disord. 2023 Jul 20;24:597. doi: 10.1186/s12891-023-06585-6 (PMC10357736; doi:10.1186/s12891-023-06585-6)
Supplement: Supplementary file 1 — Additional file 1. [file 12891_2023_6585_MOESM1_ESM.zip › MF.Gsea.1653627316242/GOMF_MODIFICATION_DEPENDENT_PROTEIN_BINDING.html]

Details for gene set GOMF\_MODIFICATION\_DEPENDENT\_PROTEIN\_BINDING[GSEA]

|  || Dataset | 55457\_collapsed\_to\_symbols.cls.cls #OA\_versus\_normal.cls.cls #OA\_versus\_normal\_repos |
| Phenotype | cls.cls#OA\_versus\_normal\_repos |
| Upregulated in class | normal |
| GeneSet | GOMF\_MODIFICATION\_DEPENDENT\_PROTEIN\_BINDING |
| Enrichment Score (ES) | -0.3085847 |
| Normalized Enrichment Score (NES) | -1.4160323 |
| Nominal p-value | 0.013975156 |
| FDR q-value | 0.25641394 |
| FWER p-Value | 1.0 |
Table: GSEA Results Summary

  

Fig 1: Enrichment plot: GOMF\_MODIFICATION\_DEPENDENT\_PROTEIN\_BINDING      
 Profile of the Running ES Score & Positions of GeneSet Members on the Rank Ordered List

  

| SYMBOL | TITLE | RANK IN GENE LIST | RANK METRIC SCORE | RUNNING ES | CORE ENRICHMENT || 1 | KAT8 | lysine acetyltransferase 8 [Source:HGNC Symbol;Acc:HGNC:17933] | 595 | 0.625 | -0.0309 | No |
| 2 | VAV1 | vav guanine nucleotide exchange factor 1 [Source:HGNC Symbol;Acc:HGNC:12657] | 601 | 0.622 | -0.0156 | No |
| 3 | PSMD4 | "proteasome 26S subunit ubiquitin receptor, non-ATPase 4 [Source:HGNC Symbol;Acc:HGNC:9561]" | 672 | 0.599 | -0.0060 | No |
| 4 | ING4 | inhibitor of growth family member 4 [Source:HGNC Symbol;Acc:HGNC:19423] | 680 | 0.598 | 0.0084 | No |
| 5 | PLCG2 | phospholipase C gamma 2 [Source:HGNC Symbol;Acc:HGNC:9066] | 725 | 0.588 | 0.0198 | No |
| 6 | BRD7 | bromodomain containing 7 [Source:HGNC Symbol;Acc:HGNC:14310] | 726 | 0.588 | 0.0345 | No |
| 7 | MLLT3 | MLLT3 super elongation complex subunit [Source:HGNC Symbol;Acc:HGNC:7136] | 764 | 0.577 | 0.0461 | No |
| 8 | DPF2 | double PHD fingers 2 [Source:HGNC Symbol;Acc:HGNC:9964] | 854 | 0.554 | 0.0531 | No |
| 9 | MINDY2 | MINDY lysine 48 deubiquitinase 2 [Source:HGNC Symbol;Acc:HGNC:26954] | 863 | 0.552 | 0.0663 | No |
| 10 | TAB2 | TGF-beta activated kinase 1 (MAP3K7) binding protein 2 [Source:HGNC Symbol;Acc:HGNC:17075] | 877 | 0.549 | 0.0791 | No |
| 11 | CBX1 | chromobox 1 [Source:HGNC Symbol;Acc:HGNC:1551] | 1331 | 0.453 | 0.0550 | No |
| 12 | CBX4 | chromobox 4 [Source:HGNC Symbol;Acc:HGNC:1554] | 1397 | 0.443 | 0.0611 | No |
| 13 | BRCC3 | BRCA1/BRCA2-containing complex subunit 3 [Source:HGNC Symbol;Acc:HGNC:24185] | 1438 | 0.437 | 0.0689 | No |
| 14 | ZBTB1 | zinc finger and BTB domain containing 1 [Source:HGNC Symbol;Acc:HGNC:20259] | 1483 | 0.431 | 0.0763 | No |
| 15 | RRP8 | ribosomal RNA processing 8 [Source:HGNC Symbol;Acc:HGNC:29030] | 1673 | 0.402 | 0.0716 | No |
| 16 | BRDT | bromodomain testis associated [Source:HGNC Symbol;Acc:HGNC:1105] | 1812 | 0.382 | 0.0704 | No |
| 17 | FAAP20 | FA core complex associated protein 20 [Source:HGNC Symbol;Acc:HGNC:26428] | 2001 | 0.361 | 0.0648 | No |
| 18 | TNIP3 | TNFAIP3 interacting protein 3 [Source:HGNC Symbol;Acc:HGNC:19315] | 2542 | 0.298 | 0.0300 | No |
| 19 | UFD1 | ubiquitin recognition factor in ER associated degradation 1 [Source:HGNC Symbol;Acc:HGNC:12520] | 2587 | 0.292 | 0.0339 | No |
| 20 | KDM8 | lysine demethylase 8 [Source:HGNC Symbol;Acc:HGNC:25840] | 2594 | 0.291 | 0.0407 | No |
| 21 | DZIP3 | DAZ interacting zinc finger protein 3 [Source:HGNC Symbol;Acc:HGNC:30938] | 2728 | 0.278 | 0.0373 | No |
| 22 | YEATS4 | YEATS domain containing 4 [Source:HGNC Symbol;Acc:HGNC:24859] | 2933 | 0.257 | 0.0278 | No |
| 23 | BABAM2 | BRISC and BRCA1 A complex member 2 [Source:HGNC Symbol;Acc:HGNC:1106] | 3002 | 0.249 | 0.0288 | No |
| 24 | TAF7 | TATA-box binding protein associated factor 7 [Source:HGNC Symbol;Acc:HGNC:11541] | 3035 | 0.246 | 0.0324 | No |
| 25 | PTPN6 | protein tyrosine phosphatase non-receptor type 6 [Source:HGNC Symbol;Acc:HGNC:9658] | 3434 | 0.208 | 0.0065 | No |
| 26 | ABRAXAS2 | "abraxas 2, BRISC complex subunit [Source:HGNC Symbol;Acc:HGNC:28975]" | 3465 | 0.205 | 0.0093 | No |
| 27 | VCP | valosin containing protein [Source:HGNC Symbol;Acc:HGNC:12666] | 3518 | 0.201 | 0.0103 | No |
| 28 | AGL | "amylo-alpha-1, 6-glucosidase, 4-alpha-glucanotransferase [Source:HGNC Symbol;Acc:HGNC:321]" | 3537 | 0.199 | 0.0139 | No |
| 29 | MORC4 | MORC family CW-type zinc finger 4 [Source:HGNC Symbol;Acc:HGNC:23485] | 4525 | 0.111 | -0.0606 | No |
| 30 | NCAPD3 | non-SMC condensin II complex subunit D3 [Source:HGNC Symbol;Acc:HGNC:28952] | 4711 | 0.095 | -0.0727 | No |
| 31 | DNAJB2 | DnaJ heat shock protein family (Hsp40) member B2 [Source:HGNC Symbol;Acc:HGNC:5228] | 4811 | 0.086 | -0.0783 | No |
| 32 | TP53BP1 | tumor protein p53 binding protein 1 [Source:HGNC Symbol;Acc:HGNC:11999] | 4946 | 0.076 | -0.0868 | No |
| 33 | CBX5 | chromobox 5 [Source:HGNC Symbol;Acc:HGNC:1555] | 4963 | 0.075 | -0.0862 | No |
| 34 | CDYL | chromodomain Y like [Source:HGNC Symbol;Acc:HGNC:1811] | 5016 | 0.072 | -0.0885 | No |
| 35 | BAG6 | BAG cochaperone 6 [Source:HGNC Symbol;Acc:HGNC:13919] | 5023 | 0.071 | -0.0871 | No |
| 36 | ING1 | inhibitor of growth family member 1 [Source:HGNC Symbol;Acc:HGNC:6062] | 5146 | 0.060 | -0.0952 | No |
| 37 | PRPF8 | pre-mRNA processing factor 8 [Source:HGNC Symbol;Acc:HGNC:17340] | 5287 | 0.049 | -0.1049 | No |
| 38 | NPLOC4 | "NPL4 homolog, ubiquitin recognition factor [Source:HGNC Symbol;Acc:HGNC:18261]" | 5303 | 0.047 | -0.1049 | No |
| 39 | ANK2 | ankyrin 2 [Source:HGNC Symbol;Acc:HGNC:493] | 5320 | 0.046 | -0.1050 | No |
| 40 | OPTN | optineurin [Source:HGNC Symbol;Acc:HGNC:17142] | 5323 | 0.046 | -0.1040 | No |
| 41 | PHF2 | PHD finger protein 2 [Source:HGNC Symbol;Acc:HGNC:8920] | 5332 | 0.045 | -0.1035 | No |
| 42 | IDE | insulin degrading enzyme [Source:HGNC Symbol;Acc:HGNC:5381] | 5491 | 0.033 | -0.1150 | No |
| 43 | MSL3 | MSL complex subunit 3 [Source:HGNC Symbol;Acc:HGNC:7370] | 5596 | 0.024 | -0.1226 | No |
| 44 | SPIN2A | spindlin family member 2A [Source:HGNC Symbol;Acc:HGNC:20694] | 5689 | 0.017 | -0.1293 | No |
| 45 | NCAPG2 | non-SMC condensin II complex subunit G2 [Source:HGNC Symbol;Acc:HGNC:21904] | 5700 | 0.016 | -0.1297 | No |
| 46 | MTF2 | metal response element binding transcription factor 2 [Source:HGNC Symbol;Acc:HGNC:29535] | 5996 | -0.004 | -0.1527 | No |
| 47 | MINDY1 | MINDY lysine 48 deubiquitinase 1 [Source:HGNC Symbol;Acc:HGNC:25648] | 6126 | -0.014 | -0.1625 | No |
| 48 | ING2 | inhibitor of growth family member 2 [Source:HGNC Symbol;Acc:HGNC:6063] | 6150 | -0.016 | -0.1639 | No |
| 49 | SQSTM1 | sequestosome 1 [Source:HGNC Symbol;Acc:HGNC:11280] | 6260 | -0.025 | -0.1718 | No |
| 50 | FAN1 | FANCD2 and FANCI associated nuclease 1 [Source:HGNC Symbol;Acc:HGNC:29170] | 6323 | -0.030 | -0.1759 | No |
| 51 | THAP7 | THAP domain containing 7 [Source:HGNC Symbol;Acc:HGNC:23190] | 6412 | -0.036 | -0.1819 | No |
| 52 | PML | PML nuclear body scaffold [Source:HGNC Symbol;Acc:HGNC:9113] | 6421 | -0.037 | -0.1816 | No |
| 53 | LYN | "LYN proto-oncogene, Src family tyrosine kinase [Source:HGNC Symbol;Acc:HGNC:6735]" | 6514 | -0.043 | -0.1877 | No |
| 54 | ING3 | inhibitor of growth family member 3 [Source:HGNC Symbol;Acc:HGNC:14587] | 6518 | -0.043 | -0.1868 | No |
| 55 | UBXN1 | UBX domain protein 1 [Source:HGNC Symbol;Acc:HGNC:18402] | 6619 | -0.051 | -0.1934 | No |
| 56 | L3MBTL1 | L3MBTL histone methyl-lysine binding protein 1 [Source:HGNC Symbol;Acc:HGNC:15905] | 6807 | -0.063 | -0.2064 | No |
| 57 | ATAD2B | ATPase family AAA domain containing 2B [Source:HGNC Symbol;Acc:HGNC:29230] | 6896 | -0.072 | -0.2115 | No |
| 58 | ZFAND6 | zinc finger AN1-type containing 6 [Source:HGNC Symbol;Acc:HGNC:30164] | 6911 | -0.073 | -0.2107 | No |
| 59 | SHARPIN | SHANK associated RH domain interactor [Source:HGNC Symbol;Acc:HGNC:25321] | 7119 | -0.090 | -0.2247 | No |
| 60 | LAPTM5 | lysosomal protein transmembrane 5 [Source:HGNC Symbol;Acc:HGNC:29612] | 7168 | -0.093 | -0.2261 | No |
| 61 | SGF29 | SAGA complex associated factor 29 [Source:HGNC Symbol;Acc:HGNC:25156] | 7363 | -0.107 | -0.2386 | No |
| 62 | MPHOSPH8 | M-phase phosphoprotein 8 [Source:HGNC Symbol;Acc:HGNC:29810] | 7413 | -0.112 | -0.2396 | No |
| 63 | PHF1 | PHD finger protein 1 [Source:HGNC Symbol;Acc:HGNC:8919] | 7429 | -0.113 | -0.2380 | No |
| 64 | RAG2 | recombination activating 2 [Source:HGNC Symbol;Acc:HGNC:9832] | 7462 | -0.115 | -0.2376 | No |
| 65 | ZZEF1 | zinc finger ZZ-type and EF-hand domain containing 1 [Source:HGNC Symbol;Acc:HGNC:29027] | 7490 | -0.118 | -0.2367 | No |
| 66 | ATRX | ATRX chromatin remodeler [Source:HGNC Symbol;Acc:HGNC:886] | 7551 | -0.123 | -0.2383 | No |
| 67 | TNIP2 | TNFAIP3 interacting protein 2 [Source:HGNC Symbol;Acc:HGNC:19118] | 7659 | -0.131 | -0.2434 | No |
| 68 | HDAC6 | histone deacetylase 6 [Source:HGNC Symbol;Acc:HGNC:14064] | 7833 | -0.143 | -0.2534 | No |
| 69 | FMR1 | FMRP translational regulator 1 [Source:HGNC Symbol;Acc:HGNC:3775] | 7944 | -0.151 | -0.2582 | No |
| 70 | CDY1 | chromodomain Y-linked 1 [Source:HGNC Symbol;Acc:HGNC:1809] | 8096 | -0.163 | -0.2659 | No |
| 71 | CARM1 | coactivator associated arginine methyltransferase 1 [Source:HGNC Symbol;Acc:HGNC:23393] | 8131 | -0.165 | -0.2644 | No |
| 72 | BRD3 | bromodomain containing 3 [Source:HGNC Symbol;Acc:HGNC:1104] | 8369 | -0.183 | -0.2784 | No |
| 73 | BRD9 | bromodomain containing 9 [Source:HGNC Symbol;Acc:HGNC:25818] | 8483 | -0.193 | -0.2824 | No |
| 74 | USP15 | ubiquitin specific peptidase 15 [Source:HGNC Symbol;Acc:HGNC:12613] | 8653 | -0.205 | -0.2905 | No |
| 75 | PWP1 | "PWP1 homolog, endonuclein [Source:HGNC Symbol;Acc:HGNC:17015]" | 8724 | -0.211 | -0.2906 | No |
| 76 | UBQLN2 | ubiquilin 2 [Source:HGNC Symbol;Acc:HGNC:12509] | 8736 | -0.211 | -0.2862 | No |
| 77 | RAD23B | "RAD23 homolog B, nucleotide excision repair protein [Source:HGNC Symbol;Acc:HGNC:9813]" | 8777 | -0.215 | -0.2839 | No |
| 78 | KMT2A | lysine methyltransferase 2A [Source:HGNC Symbol;Acc:HGNC:7132] | 8807 | -0.217 | -0.2807 | No |
| 79 | MSH6 | mutS homolog 6 [Source:HGNC Symbol;Acc:HGNC:7329] | 8846 | -0.221 | -0.2782 | No |
| 80 | ZCWPW1 | zinc finger CW-type and PWWP domain containing 1 [Source:HGNC Symbol;Acc:HGNC:23486] | 8865 | -0.222 | -0.2740 | No |
| 81 | DAXX | death domain associated protein [Source:HGNC Symbol;Acc:HGNC:2681] | 8926 | -0.226 | -0.2730 | No |
| 82 | ZMYND11 | zinc finger MYND-type containing 11 [Source:HGNC Symbol;Acc:HGNC:16966] | 8952 | -0.228 | -0.2692 | No |
| 83 | ZZZ3 | zinc finger ZZ-type containing 3 [Source:HGNC Symbol;Acc:HGNC:24523] | 8991 | -0.231 | -0.2664 | No |
| 84 | RAD23A | "RAD23 homolog A, nucleotide excision repair protein [Source:HGNC Symbol;Acc:HGNC:9812]" | 9028 | -0.234 | -0.2634 | No |
| 85 | TDRD3 | tudor domain containing 3 [Source:HGNC Symbol;Acc:HGNC:20612] | 9080 | -0.238 | -0.2614 | No |
| 86 | EPS15 | epidermal growth factor receptor pathway substrate 15 [Source:HGNC Symbol;Acc:HGNC:3419] | 9409 | -0.265 | -0.2804 | No |
| 87 | PYGO1 | pygopus family PHD finger 1 [Source:HGNC Symbol;Acc:HGNC:30256] | 9628 | -0.286 | -0.2903 | No |
| 88 | IKBKE | inhibitor of nuclear factor kappa B kinase subunit epsilon [Source:HGNC Symbol;Acc:HGNC:14552] | 9646 | -0.287 | -0.2844 | No |
| 89 | TRIM24 | tripartite motif containing 24 [Source:HGNC Symbol;Acc:HGNC:11812] | 9927 | -0.314 | -0.2984 | No |
| 90 | SMARCA4 | "SWI/SNF related, matrix associated, actin dependent regulator of chromatin, subfamily a, member 4 [Source:HGNC Symbol;Acc:HGNC:11100]" | 10009 | -0.322 | -0.2966 | No |
| 91 | MORC3 | MORC family CW-type zinc finger 3 [Source:HGNC Symbol;Acc:HGNC:23572] | 10037 | -0.325 | -0.2906 | No |
| 92 | CBX2 | chromobox 2 [Source:HGNC Symbol;Acc:HGNC:1552] | 10065 | -0.327 | -0.2845 | No |
| 93 | UBQLN3 | ubiquilin 3 [Source:HGNC Symbol;Acc:HGNC:12510] | 10072 | -0.328 | -0.2767 | No |
| 94 | DNAJC2 | DnaJ heat shock protein family (Hsp40) member C2 [Source:HGNC Symbol;Acc:HGNC:13192] | 10081 | -0.329 | -0.2691 | No |
| 95 | SUZ12 | SUZ12 polycomb repressive complex 2 subunit [Source:HGNC Symbol;Acc:HGNC:17101] | 10104 | -0.331 | -0.2625 | No |
| 96 | PSME4 | proteasome activator subunit 4 [Source:HGNC Symbol;Acc:HGNC:20635] | 10694 | -0.392 | -0.2987 | Yes |
| 97 | PHIP | pleckstrin homology domain interacting protein [Source:HGNC Symbol;Acc:HGNC:15673] | 10715 | -0.395 | -0.2904 | Yes |
| 98 | GLYR1 | glyoxylate reductase 1 homolog [Source:HGNC Symbol;Acc:HGNC:24434] | 10807 | -0.406 | -0.2873 | Yes |
| 99 | CXXC1 | CXXC finger protein 1 [Source:HGNC Symbol;Acc:HGNC:24343] | 10853 | -0.411 | -0.2805 | Yes |
| 100 | TTLL12 | tubulin tyrosine ligase like 12 [Source:HGNC Symbol;Acc:HGNC:28974] | 10861 | -0.411 | -0.2707 | Yes |
| 101 | KDM4A | lysine demethylase 4A [Source:HGNC Symbol;Acc:HGNC:22978] | 10936 | -0.423 | -0.2659 | Yes |
| 102 | IKBKG | inhibitor of nuclear factor kappa B kinase regulatory subunit gamma [Source:HGNC Symbol;Acc:HGNC:5961] | 10986 | -0.428 | -0.2590 | Yes |
| 103 | KDM5A | lysine demethylase 5A [Source:HGNC Symbol;Acc:HGNC:9886] | 11097 | -0.443 | -0.2564 | Yes |
| 104 | CBX3 | chromobox 3 [Source:HGNC Symbol;Acc:HGNC:1553] | 11113 | -0.445 | -0.2464 | Yes |
| 105 | PHF8 | PHD finger protein 8 [Source:HGNC Symbol;Acc:HGNC:20672] | 11592 | -0.514 | -0.2709 | Yes |
| 106 | CHD5 | chromodomain helicase DNA binding protein 5 [Source:HGNC Symbol;Acc:HGNC:16816] | 11776 | -0.545 | -0.2716 | Yes |
| 107 | RBBP5 | "RB binding protein 5, histone lysine methyltransferase complex subunit [Source:HGNC Symbol;Acc:HGNC:9888]" | 11816 | -0.553 | -0.2607 | Yes |
| 108 | CHD8 | chromodomain helicase DNA binding protein 8 [Source:HGNC Symbol;Acc:HGNC:20153] | 11825 | -0.554 | -0.2474 | Yes |
| 109 | BPTF | bromodomain PHD finger transcription factor [Source:HGNC Symbol;Acc:HGNC:3581] | 11870 | -0.565 | -0.2367 | Yes |
| 110 | UIMC1 | ubiquitin interaction motif containing 1 [Source:HGNC Symbol;Acc:HGNC:30298] | 11919 | -0.576 | -0.2259 | Yes |
| 111 | JARID2 | jumonji and AT-rich interaction domain containing 2 [Source:HGNC Symbol;Acc:HGNC:6196] | 12030 | -0.600 | -0.2195 | Yes |
| 112 | YEATS2 | YEATS domain containing 2 [Source:HGNC Symbol;Acc:HGNC:25489] | 12179 | -0.639 | -0.2150 | Yes |
| 113 | MBTD1 | mbt domain containing 1 [Source:HGNC Symbol;Acc:HGNC:19866] | 12189 | -0.641 | -0.1996 | Yes |
| 114 | ZMYND8 | zinc finger MYND-type containing 8 [Source:HGNC Symbol;Acc:HGNC:9397] | 12214 | -0.649 | -0.1852 | Yes |
| 115 | RNF31 | ring finger protein 31 [Source:HGNC Symbol;Acc:HGNC:16031] | 12332 | -0.686 | -0.1771 | Yes |
| 116 | KDM7A | lysine demethylase 7A [Source:HGNC Symbol;Acc:HGNC:22224] | 12390 | -0.704 | -0.1639 | Yes |
| 117 | OTUD7B | OTU deubiquitinase 7B [Source:HGNC Symbol;Acc:HGNC:16683] | 12539 | -0.767 | -0.1562 | Yes |
| 118 | SPIN1 | spindlin 1 [Source:HGNC Symbol;Acc:HGNC:11243] | 12559 | -0.777 | -0.1381 | Yes |
| 119 | CBX8 | chromobox 8 [Source:HGNC Symbol;Acc:HGNC:15962] | 12586 | -0.787 | -0.1204 | Yes |
| 120 | BRD4 | bromodomain containing 4 [Source:HGNC Symbol;Acc:HGNC:13575] | 12606 | -0.800 | -0.1018 | Yes |
| 121 | TNFAIP3 | TNF alpha induced protein 3 [Source:HGNC Symbol;Acc:HGNC:11896] | 12631 | -0.814 | -0.0832 | Yes |
| 122 | BRD2 | bromodomain containing 2 [Source:HGNC Symbol;Acc:HGNC:1103] | 12709 | -0.871 | -0.0674 | Yes |
| 123 | CHD1 | chromodomain helicase DNA binding protein 1 [Source:HGNC Symbol;Acc:HGNC:1915] | 12745 | -0.908 | -0.0473 | Yes |
| 124 | BAZ2A | bromodomain adjacent to zinc finger domain 2A [Source:HGNC Symbol;Acc:HGNC:962] | 12879 | -1.166 | -0.0284 | Yes |
| 125 | TAF1 | TATA-box binding protein associated factor 1 [Source:HGNC Symbol;Acc:HGNC:11535] | 12881 | -1.192 | 0.0015 | Yes |
Table: GSEA details [plain text format]

  

Fig 2: GOMF\_MODIFICATION\_DEPENDENT\_PROTEIN\_BINDING      
 Blue-Pink O' Gram in the Space of the Analyzed GeneSet

  

Fig 3: GOMF\_MODIFICATION\_DEPENDENT\_PROTEIN\_BINDING: Random ES distribution      
 Gene set null distribution of ES for **GOMF\_MODIFICATION\_DEPENDENT\_PROTEIN\_BINDING**
